# Let’s talk about standards: a commentary on standards of practice in empirical bioethics

**DOI:** 10.1186/s12910-018-0303-4

**Published:** 2018-07-11

**Authors:** Alan Cribb

**Affiliations:** King’s College London, London, UK

**Keywords:** Empirical bioethics, Standards, Consensus, Disagreement

## Abstract

This commentary welcomes the work of Ives et al. on Standards of practice in Empirical Bioethics, and especially the dialogical spirit in which the standards have been constructed and offered. It also raises some questions about the consistent interpretation and use of such standards.

## Main text

Surely most people are ambivalent about standards. We wish to set and keep standards, of course, but we are conscious that standards can go wrong in various ways, for example: they can be badly calibrated, including by being too vague or too narrowly defined; or they can have unintended consequences such as by having oppressive effects when they are meant to be helpful. I may wish to be respectful towards my parents but the very act of being reminded of that standard can cause me to fail.

The authors of “Standards of practice in Empirical Bioethics research” [1] deserve many congratulations not only for recognising that standard setting is a tricky business but for conscientiously striving to keep their balance whilst navigating these hazardous paths.

The whole work is presented as a staging post in a move ‘towards’ consensus about standards. And the results are explicitly treated as both the consequence of one process and the start of another one – they reflect dialogue and debate amongst the 16 participants and co-authors and they are being offered in order to initiate a larger conversation. The resulting text wonderfully holds together two sets of tensions between closing things down and opening them up. It carefully articulates the consensus statements whilst clarifying the disagreements that had to be negotiated in the process of formulating them. And it also embodies a respectful dialogue with the community of readers – “This specifies where we got to. But what do you think?”

Because so much is going on in this richly woven text it is impossible to respond adequately in a short commentary. But I will just make some brief observations relating to each of the two phases of dialogue – the process of standard setting reported in the paper and the anticipated phase of standard reception or use.

The first thing that becomes manifest on reading the text is that finding a framing, and a language, for standards is far from straightforward. This is not surprising given that the project of empirical bioethics centres around, and depends upon, foundational debates about the nature of knowledge and ethics. When the issues in question are at this foundational level then every word that is chosen can have different implications and resonances for different people. This applies, not least, to the central business of capturing what is going on when the word ‘empirical’ is added to the word ‘bioethics’. Unless we are happy to extend the label of ‘empirical bioethics’ to essentially descriptive work (and I will come back to that idea) it seems uncontroversial to say that empirical bioethics is a field in which the methods of empirical social sciences and a concern for what ought to be done co-exist. However the reported discussion and disagreements around, and the resultant and very measured wording of, standards 2 and 4 show how difficult it is to definitely say much more than this. These standards (along with 5 and 6) refer to the business of ‘integration’. Standard 2 talks about the integration of ‘empirical methods’ and ‘ethical argument’; standard 4 sees integration as requiring an account of how ‘the empirical’ and ‘the normative’ are related. The commentary provided makes it very clear that – even if we are merely seeking to do justice to the participants in the consensus seeking process - we should interpret the idea of ‘integration’ in an elastic way. Specifically we are not being asked to presume either that empirical and normative kinds of inquiry can be seamlessly combined or that they represent two clearly distinct kinds of things in the first place. Those who are unhappy with the latter presumption may, as the authors note, be resistant to engaging with the concept of integration, seeing it as “the construct of an artificial philosophical separation”. In short, a word that sits at the core of the standards - ‘integration’ – albeit quite explicitly allowing for “an expansive understanding of ‘integration’” – seems problematic, for fundamental reasons, to some of the participants in the consensus setting process. This raises questions about how far such standards would be accepted, interpreted and applied in consistent ways, especially when they are dislodged from the narrative within which they are presented.

The (unavoidable) foundational disputes are managed by the authors – including in the formulation of the standards themselves – with skill and subtlety. For example, the standards require researchers simply to articulate their broad epistemological/ meta-ethical position but to explain and justify how this position is translated into specific project methodology. This means that researchers are not expected to try and persuade their readers of the validity of their underlying philosophical stance (although they will typically be signposting readers to currents of literature where that is attempted) but they are expected to make that stance sufficiently clear as part of enabling the reader to make sense of, and assess the appropriateness and rigour of, the specific conjunction of aims and methods set out. In clarifying and defending these requirements the authors explain that “it would be unreasonably burdensome to expect researchers to defend all meta-ethical and epistemological commitments and assumptions in every paper”.

I would like, as an aside, to briefly note the wisdom of this remark about burdensomeness. I would guess that many people who think of themselves as doing empirical bioethics will not only be pleased not to have to constantly “defend all meta-ethical and epistemological commitments and assumptions” but will be cautious about their capacity to defend them at all (if ‘defend’ implies an effort that is sufficiently sophisticated to be a credible candidate for success rather than simply one of ‘taking a stab’). The people I have in mind here are not indifferent to the demands of philosophical ethics but, rather, are mindful of just how onerous those demands are.

Turning to the question about the potential reception and use of the standards then what should we imagine about who might make use of them, and when, where and how? In opening up the conversation these are some of the key questions the authors are raising for readers. The authors have done a lot of work and much of the rest is for other people to debate and fathom. I have already raised a concern about the fundamental challenge of different users interpreting the standards in diverse ways. But, leaving that aside, it is clear that there are a number of other uncertainties and complications to address. I will just flag up one practical uncertainty here – assuming we were to agree standards for empirical bioethics research (along the lines of the ones recommended here) then to what research efforts and what fractions of efforts should these standards be applied? The authors are sensitive to the danger that the standards will be seen as drawing territorial boundaries between ‘real’ empirical bioethics (as defined by the standards) and other things calling themselves empirical bioethics. But, as they make clear, this is inevitable given their project – a necessary part of specifying standards for ‘x’ is specifying what is meant by ‘x’. The paper explicitly lists four kinds of research that are not covered by the standards but that, at least in some cases, might be thought by some people to fall within a broader notion of empirical bioethics. The upshot of this is that different strands of research within a large research programme, and sometimes with a single project, will fall either side of this boundary. Even assuming that some researchers accept the standards reported in this paper they may find it more comfortable to refer to the whole programme or project as work in ‘empirical bioethics’ (by virtue of it containing some clear strand that fits the standards specification) rather than to disaggregate the programme or project into parts. People have to find convenient summary labels for their work and although not everything can be allowed to be assigned any label the limits of what counts as defensible ‘packaging claims’ are fuzzy. Perhaps more significantly the same applies if we ‘zoom in’ from programmes and projects to specific research activities and products, and to elements of activities and products (e.g. individual papers). Obviously not every single research breath or every clause of every sentence has to conform to whatever standards are agreed – this is obviously a nonsense. Some notion of a suitably sized ‘unit of research’ is entailed in the interpretation and application of these standards and that may call for further thought. This is not meant as an academic point but a practical one – for example, when is it OK to publish papers that do not explain and justify project methodology if such an account exists somewhere else? Given the notorious challenges of juggling the demands of journals in interdisciplinary fields some pragmatic flexibility in the overt application of standards is no doubt needed.

This whole intervention is to be applauded. The authors offer us some standards and invite us to a conversation – both these things seem welcome to me. Time will tell how valuable the standards themselves are but, I would suggest, the dialogical spirit of the invitation is invaluable.
